# Left-Sided Acutely Irreducible Terminal Ileum Hernia With Cecum, Appendix, and Sliding Sigmoid: Case Report and Review of the Literature

**DOI:** 10.7759/cureus.79591

**Published:** 2025-02-24

**Authors:** Fahad Alshubaily, Jumana A Fatani, Abdullah A Alqarawi, Suliman Aldubayan

**Affiliations:** 1 General Ssurgery, King Saud Medical City, Riyadh, SAU; 2 Surgery, Specialized Medical Center, Riyadh, SAU; 3 General Surgery, King Saud Medical City, Riyadh, SAU

**Keywords:** amyand’s hernia, appendix, cecum, hernia, incarcerated, inguinal, mesh, sigmoid, sliding, strangulated

## Abstract

Amyand's hernia is the presence of the vermiform appendix within the inguinal hernia sac, while a sliding hernia involves the herniation of a retroperitoneal organ outside the abdominal cavity. The hernia sac can contain various intraabdominal organs, including the small bowel, cecum, appendix, omentum, or ovary and fallopian tube. A hernial sac containing the appendix on the left side is rare, and the presence of other organs in combination is also uncommon. We are presenting a case of a 45-year-old male who presented with irreducible left inguinal swelling and obstructing symptoms. Interestingly, a large left indirect hernia sac was seen intraoperatively containing incarcerated cecum, terminal ileum, appendix, and sliding sigmoid colon. Left-sided incarcerated terminal ileum, cecum, appendix, and sliding hernia can be challenging because the symptoms are often nonspecific. However, surgery is often the only way to definitively diagnose these types of hernia.

## Introduction

Amyand's hernia, named after Claudius Amyand, who first described it in 1735 [1-3], is characterized by the presence of the appendix within the inguinal hernia sac. During a herniotomy, Amyand encountered an inflamed appendix in the hernia sac and subsequently performed an appendectomy through the same incision [1,3]. While hernial sacs containing the appendix are generally rare, their occurrence on the left side is even more uncommon [1,3]. Diagnosing Amyand's hernia can be challenging, and it is often not discovered until surgery is performed [2]. Conversely, a sliding hernia involves the protrusion of a retroperitoneal organ outside the abdominal cavity [4]. Any intraabdominal organ can be encountered in the hernia sac, including the small bowel, cecum, appendix, omentum, or ovary and fallopian tube [1,2,5].

## Case presentation

**Figure 1:**
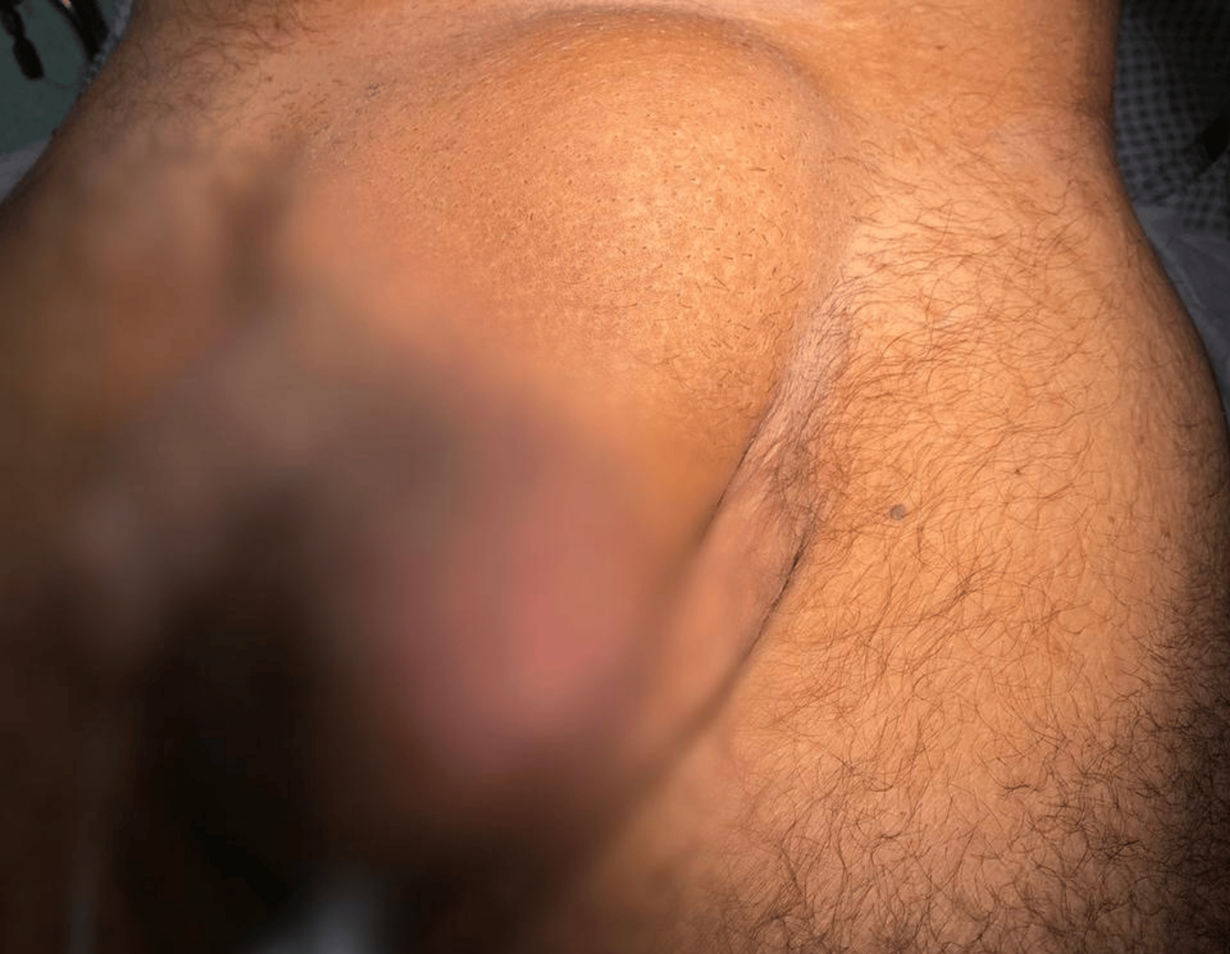
Left sided incarcerated inguinoscrotal hernia

**Figure 2:**
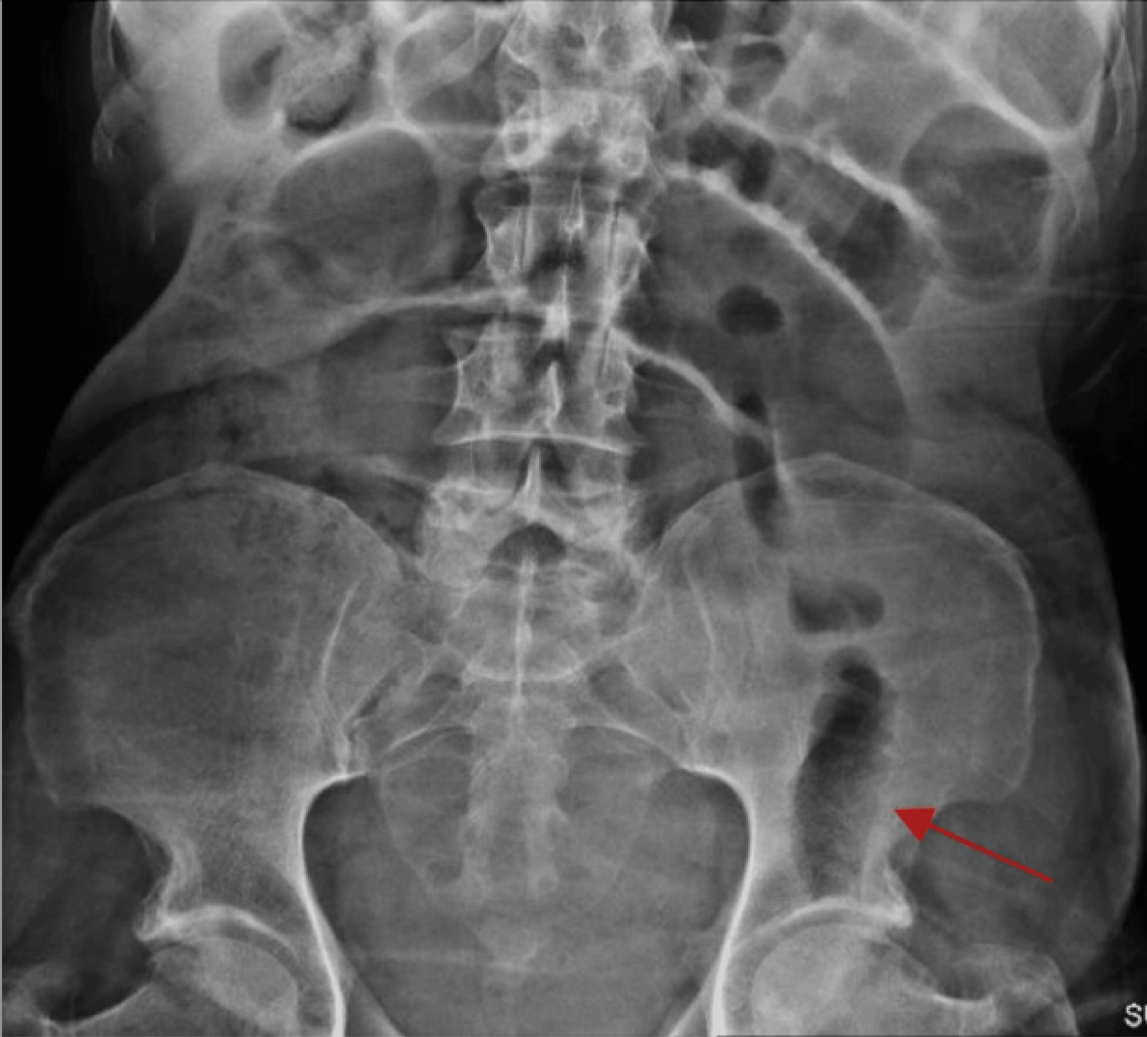
Abdominal X-ray showing small bowel on the left lower quadrant (red arrow)

**Figure 3:**
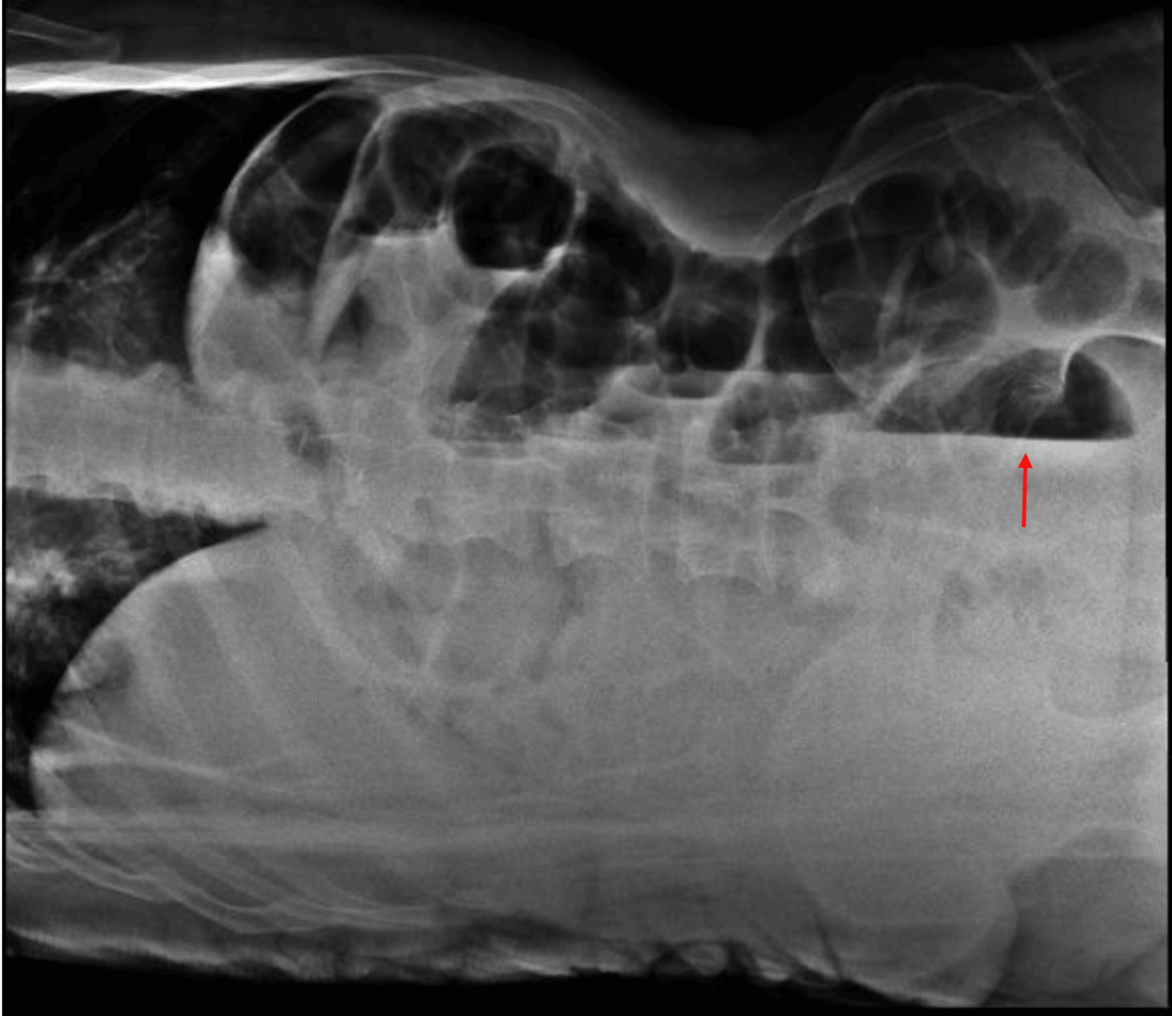
Abdominal X-ray showing air fluid levels at different levels (red arrow showing air fluid level)

**Figure 4:**
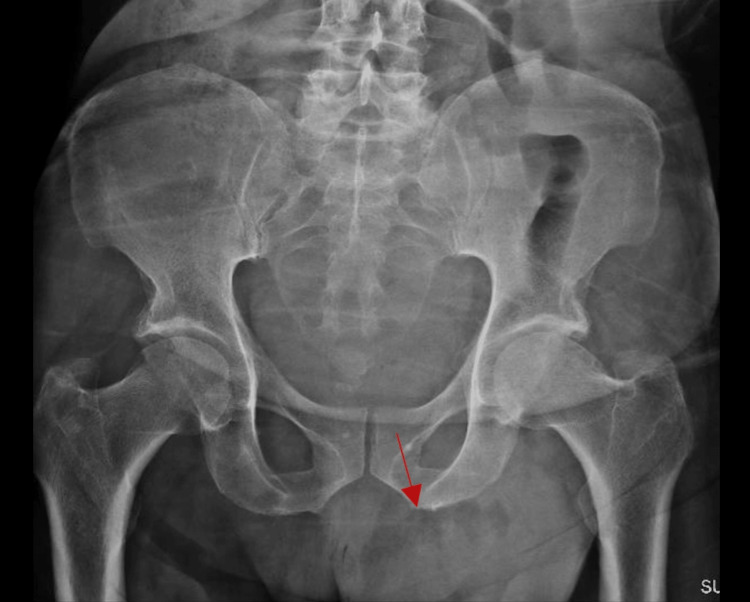
Pelvic X-ray showing small bowel in the left inguinal area (red arrow)

**Figure 5:**
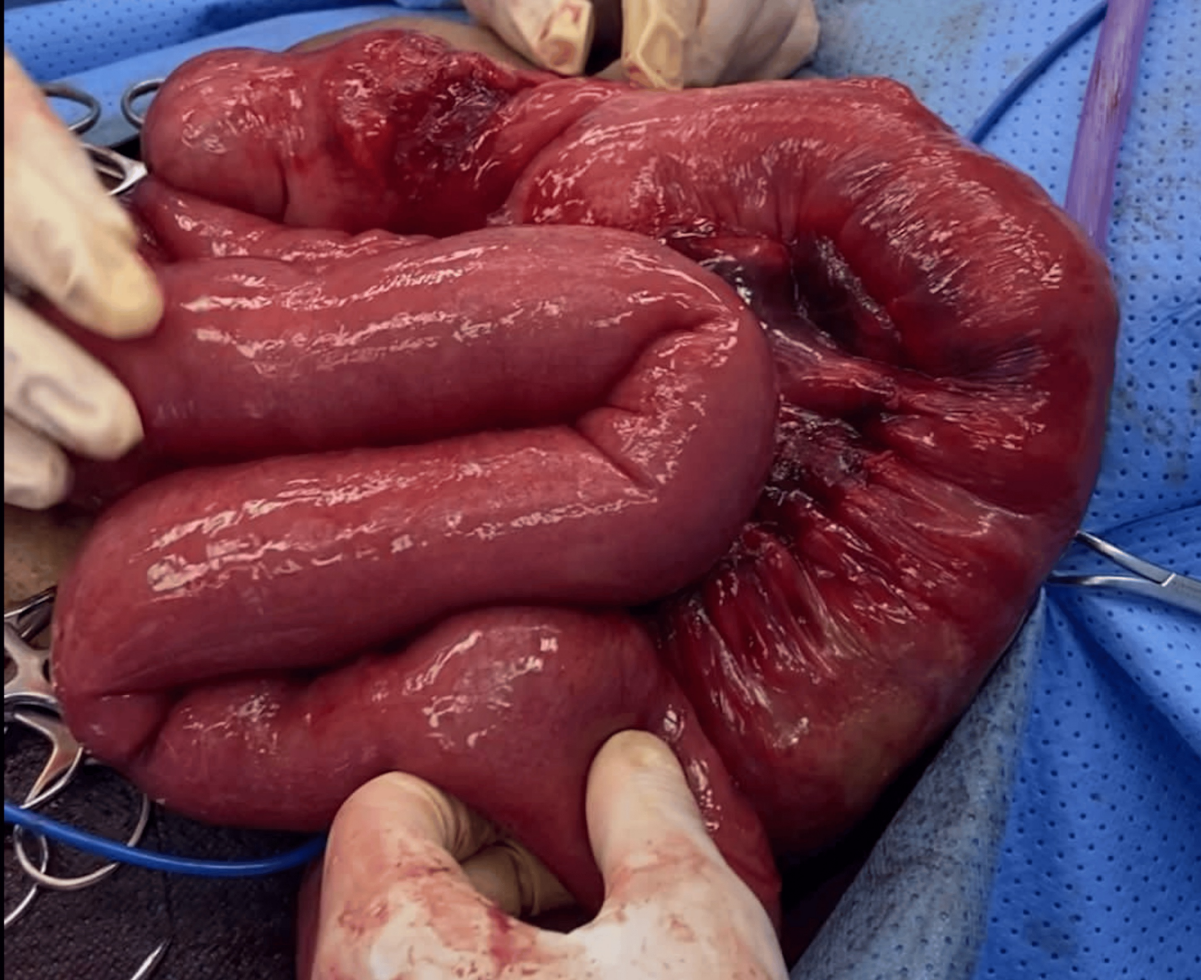
Incarcerated terminal ileum with mesenteric hematoma

**Figure 6:**
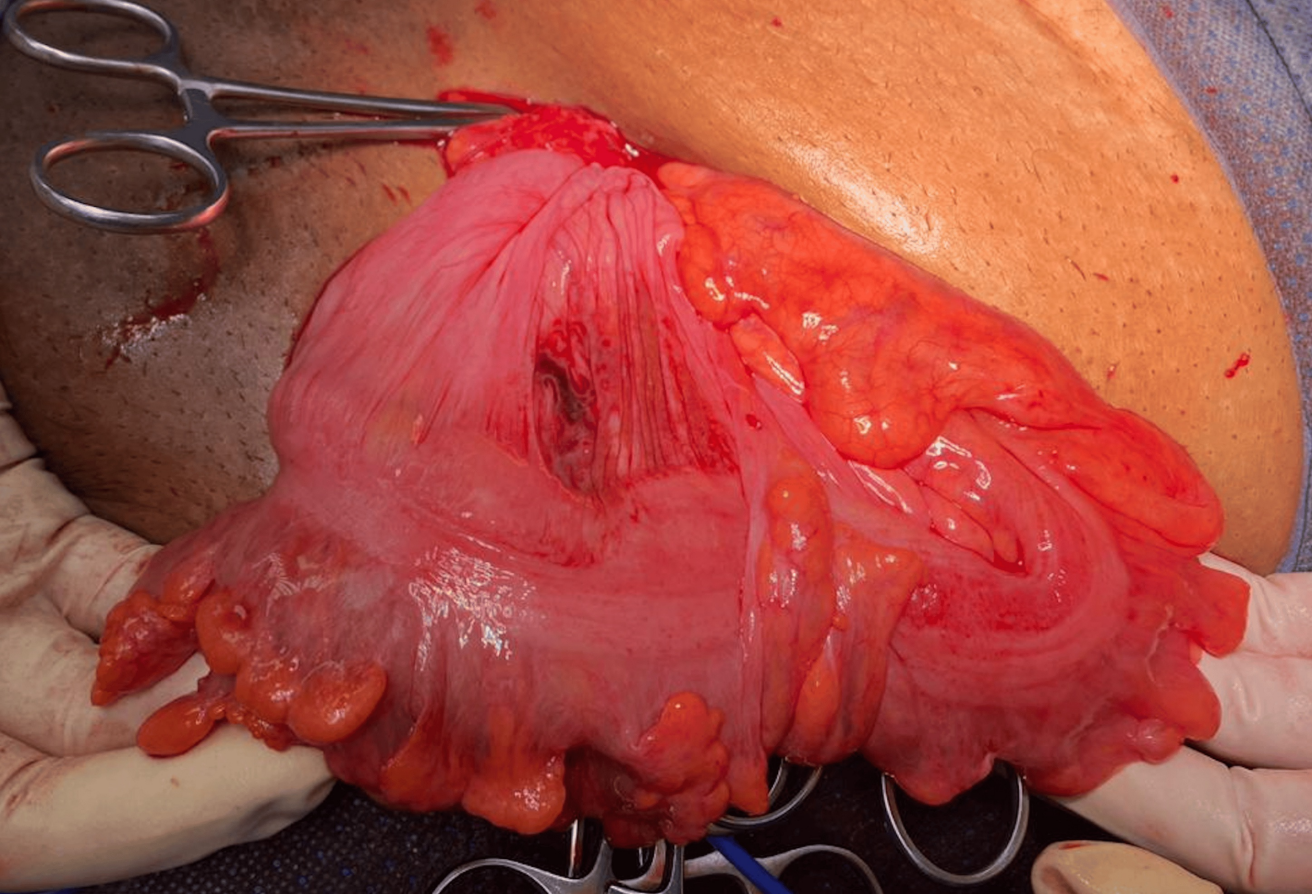
Sliding healthy sigmoid

A 45-year-old male with a past medical history of diabetes mellitus and hypertension and a past surgical history of sleeve gastrectomy that was done three years ago and a left open donor nephrectomy 20 years ago presented to the emergency department with a left inguinal swelling for two years which became irreducible four days prior to presentation, associated with nausea and vomiting, as well as obstipation for two days. On examination, an irreducible left indirect inguinal hernia was identified (Figure 1) with no cough impulse and was tender to touch. Abdominal and pelvic X-ray was done, which showed a small bowel loop in the left inguinal region with a dilated small bowel and multiple air-fluid levels (Figures 2-4). He was shifted emergently to the operating room, as in the case of left incarcerated indirect inguinal hernia. Through an open left inguinal incision, a large indirect hernia sac was seen containing incarcerated and dusky cecum, terminal ileum, and appendix with multiple mesenteric hematomas and around 100 ccs of turbid fluid came out, as well as sliding healthy sigmoid colon (Figures 5, 6). After hot-soaked saline gauze and 100% FIo2, the bowel became normal with a good pulse and good peristalsis. Contents were reduced, and cord and Vas deference were seen and preserved. The sac was excised and ligated. The internal ring was refashioned and repaired by Lytle repair, with the floor reinforced by Bassini repair. The patient was discharged two days after the surgery in good condition. The patient was seen in the outpatient clinic for a follow-up and was doing well.

## Discussion

The incidence of Amyand's hernia is still not definitively established, but various studies have reported it to be within the range of 0.19% to 1.7% [3,6]. In adult patients specifically, the reported incidence falls within 0.28% to 1% [3]. Although hernial sacs containing the appendix are generally uncommon, their occurrence on the left side is even rarer [1, 3]. The right-sided Amyand's hernias are statistically more prevalent than the left-sided. This is largely attributed to the anatomical positioning of the appendix, typically found on the right side of the abdomen [2,7]. Amyand's hernias are more frequently observed in males and can occur across all age groups, from premature infants to elderly individuals [2]. However, children are three times more prone to developing this condition compared to adults, primarily due to the incomplete obliteration of the processus vaginalis in their developing bodies [3,8].

The mechanism behind Amyand's hernia is still not well established [6]. However, several factors have been linked to an increased risk of developing left-sided Amyand's hernia, including malrotation of the intestine, increased cecum mobility, situs inversus, and an abnormally long appendix [1,3,8]. Cecum mobility is due to the failure of the right colonic mesentery attaching to the lateral peritoneum, leading to an unattached cecum and ascending colon that are free to rotate [3]. Left-sided incarceration is much less common than right-sided incarceration [7]. Diagnosing Amyand's hernia can be challenging and often goes undetected until surgery is performed [2,6]. During surgery, the hernia can contain various gastrointestinal and pelvic organs such as the appendix, cecum, ileum, and others, even in cases of left-sided hernias [2]. While preoperative diagnosis of Amyand's hernia can be achieved through imaging techniques like ultrasound (US) or abdominal computed tomography (CT) scan, it is not typically recommended for this specific condition [3]. Nonetheless, some authors have mentioned the use of CT with contrast and contrast enema to identify the presence of the ileum, cecum, and/or appendix in left-sided Amyand's hernias in adults [2,6]. Additionally, they may also reveal the malrotation of the cecum and situs inversus [7]. In cases where incarceration is suspected, immediate surgical intervention is necessary without the need for imaging confirmation [6].

Patients usually exhibit symptoms of acute appendicitis and/or complicated hernia, such as obstruction or strangulation [7,8]. When strangulation or perforation does not occur, patients typically present with nonspecific symptoms of an inguinal hernia [3]. An 83% incidence of a painful inguinal or inguinoscrotal mass has been reported [6]. Often, a physical examination is adequate to prompt emergency operations in the case of an incarcerated hernia [2]. Most left-sided Amyand's hernias are treated with herniorrhaphy with or without an appendectomy and repair of any serosal tears [3]. Laparoscopic repair for non-strangulated Amyand's hernias has been reported [3]. Performing an appendectomy increases the risk of surgical site infection and recurrence of the hernia due to the need for a larger incision [3]. In cases of appendicitis or peritonitis, mesh placement is not recommended because of the increased risk of sepsis [3]. Management of a noninflamed appendix in a left inguinal hernia is still controversial [9]. Some believe appendectomy should not be performed as it converts the surgery from clean to clean contaminated, which increases the risk of wound and mesh infection [6,9]. On the other hand, others think an appendectomy is necessary for a left-sided Amyand's hernia to prevent potential future appendicitis, given its potential for an atypical clinical presentation [9]. However, the occurrence of sliding hernias constitutes 6-8% of all elective inguinal hernia repairs [4]. A sliding hernia is commonly observed in infant females, accounting for up to 20% of all hernias, with the contents typically involving the fallopian tube and/or ovary [4]. In adults, sliding hernias predominantly occur in males. Similar to Amyand's hernia, a sliding hernia can involve any abdominal organ within the hernia sac, such as part of the omentum or small or large bowels [5]. While the cecum, appendix, and ascending colon are often found on the right side, the sigmoid colon is frequently noted to protrude through abdominal defects in the left inguinal region as a sliding hernia due to anatomical considerations [5]. Identifying a sliding hernia preoperatively is challenging as there are no specific signs indicating the likelihood of a sliding hernia [4]. Elderly patients with large hernias who present a prolonged history of an inguinal lump are at higher risk of having a retroperitoneal organ protruding into the hernia sac [4]. The diagnosis is typically confirmed intraoperatively when the hernia sac is opened [4]. Incarceration occurs in almost 10% of all inguinal hernias, and the placement of mesh in such cases is a matter of debate due to the risk of mesh infection [5]. Table 1 presents a summary of adult left Amyand's hernia cases. In the literature, there are 14 reported cases of left-sided incarcerated or strangulated Amyand's hernia. Among these cases, 12 out of 14 involved the cecum found in the hernial sac along with the appendix. Four cases included the terminal ileum, cecum, and the appendix. Notably, there were no reported cases involving a sliding sigmoid with the terminal ileum, cecum, and appendix. The treatment for all cases involved open repair, except for one case where open repair was initiated followed by diagnostic laparoscopy. Exploratory laparotomy was done for some cases that needed further resection. The documented causes of the hernias were cecum mobility in most cases, with one case involving cecum mobility with spine side-bending deformity and one case involving nonrotation of the bowel. Our case suggests that cecum mobility is the most likely cause of this condition. Initial diagnoses for 11 cases were incarcerated hernias, with the remaining case diagnosed as a strangulated or obstructed hernia. Additionally, two cases were diagnosed as a strangulated hernia during the operation. Two cases have recurrence on the same site of the previous hernia repair.

**Table 1:** Summary or reported cases in the literature

Article	Age	Gender	Presentation	Image	Type	Surgery	Contents	Cause
Ghafouri et al., 2012 [1]	60	M	Left scrotal mass	-	Incarcerated indirect hernia	Herniorrhaphy + Lichtenstein repair	Cecum, appendix	-
Vuu et al., 2018 [3]	76	F	Acute abdomen	CT: Small bowel proximal to the terminal ileum within the left inguinal ring Multiple loops of small bowel demonstrated decreased wall enhancement with ischemic changes.	Strangulated hernia	Sac resected, hernia reduced and necrotic bowel resected, primary repair, ileo-ascending colon anastomosis	Distal ileum, appendix, cecum, Cecal bascule	Mobile cecum
Corvatta et al., 2023 [6]	72	F	Abdominal pain and nausea	US: Protrusion of a hollow viscus through a 42 mm fascial continuum.	Incarcerated inguinal hernia	Large direct hernial sac and a synchronous ipsilateral femoral hernia primary closure of the posterior wall defect and the femoral ring by primary McVay repair with placement of a polypropylene mesh	Cecum, appendix	Mobile cecum
Unver et al., 2013 [7]	32	M	Irreducible inguinal mass with pain, nausea, and vomiting	CT: Mobile cecum switched to the left side of the abdomen, with co-existing inflammatory echogenic findings and a left side inguinal hernia sac including appendix vermiformis	Incarcerated recurrent left hernia	Appendectomy and repaired internal ring with primary sutures	Appendix vermiformis	Mobile cecum
Nowrouzi et al., 2021 [8]	62	M	Lower abdominal pain, nausea and constipation	CT: Large left-sided inguinal hernia with possible bowel strangulation involving the colon	Incarcerated recurrent left inguinal hernia “Pantaloon hernia”	Started with an open inguinal incision then diagnostic laparoscopy large hernia defect was repaired with a polypropylene mesh	Omentum, a loop of transverse colon, entire cecum, appendix	-
Dong et al., 2014 97]	63	M	Painful left groin lump, nausea, vomiting and obstipation	CT: Ileocecum within the left inguinoscrotal sac	Incarcerated indirect hernia	Appendectomy and left herniorrhaphy with biological mesh	Cecum, portion of ascending colon, distal terminal ileum, inflamed appendix	Mobile cecum and spine side-bending deformity
Mongardini et al., 2015 [10]	68	M	Inguinal scrotal pain and fever	-	Incarcerated inguinal hernia	Abscess drainage Debridement of the herniated organs + appendectomy Resection of necrotic omentum Hernioplasty according to Postempski technique	Abscess, phlegmonous perforated appendix, cecum, ascending colon, last ileal loops and bladder	-
Turanlı et al., 2011 [11].	54	M	Left groin pain	US: 10 cm in length inactive, edematous intestinal section within the inguinal hernia	Incarcerated inguinal hernia	Appendectomy and primary hernia repair	Inflamed vermiform appendix	-
Breitenstein et al., 2005 [12]	81	F	Left groin pain	US: incarcerated left-side inguinal hernia	Incarcerated indirect hernia	Appendectomy + Shouldice repair	Part of cecum, incarcerated vermiform appendix	-
Maeda et al., 2014 [13]	62	M	Large left inguinoscrotal hernia Right inguinal hernia	CT: Dislocation of the ileum, appendix, cecum, and ascending colon into the left-sided inguinoscrotal hernial sac	Incarcerated hernia	Hernial repair	Ileum, appendix, cecum, ascending colon	-
Ravishankaran et al., 2013 [14]	35	M	Obstructed left inguinal hernia	X-ray: Dilated small bowel loops	Incarcerated indirect hernia	Herniorraphy + appendectomy + gangrenous omentum excised	Gangrenous omentum, small bowel, appendix	Non-rotation of the bowel
Malik et al., 2012 [15]	64	M	Fever, pain, vomiting, irreducible left inguinal hernia	-	Strangulated irreducible indirect inguinal hernia	Hernial repair + limited right hemicolectomy with ileocolic anastomosis	Gangrenous cecum and appendix	Mobile cecum
Johari et al., 2009 [16]	70	M	Not mentioned	-	Not mentioned	Herniorraphy + appendectomy	Cecum, appendix	-
Tayade et al., 2008 [17]	34	M Explore	Left groin pain	-	Incarcerated hernia	Hernia repair + quartercolectomy with ileo-ascending colon anastomosis	Vermiform appendix and patch of gangrenous wall of cecum	Mobile cecum

## Conclusions

Left-sided Amyand's hernia and sliding hernia can be quite difficult to diagnose and often go undetected until surgery is performed. Patients usually present with symptoms of acute appendicitis and/or obstructed or strangulated hernia. A high index of suspicion should be considered as the differential diagnosis can include an incarcerated or strangulated hernia, acute appendicitis, or other emergencies. When incarceration is suspected, prompt surgical intervention is required, even without imaging confirmation. Here, we presented a case of a left-sided incarcerated hernia containing cecum, terminal ileum, appendix, and sliding sigmoid that was managed through the inguinal incision.
